# Ebola Virus Diseases in Africa: a commentary on its history, local and global context

**DOI:** 10.11694/pamj.supp.2015.22.1.6652

**Published:** 2015-10-11

**Authors:** Paschal Kum Awah, Alphonse Um Boock, Kaiseuh Awah Kum

**Affiliations:** 1 Department of Anthropology, Faculty of Arts, Letters and Social Sciences, University of Yaounde I, PO Box 755, Yaounde, Cameroon; 2 Center for Population Studies and Health Promotion, PO Box 7535, Yaounde-Cameroon; 3 Regional Representative, Fairmed Foundation, Africa, Yaounde, Cameroon; 4 Department of Psychology, Faculty of Arts, Letters and Social Sciences, University of Yaounde I, PO Box 755, Yaounde, Cameroon

**Keywords:** Ebola Virus Disease, neglect, West Africa, African model, culture

## Abstract

Ebola Virus Disease (EVD) started as a minor infection in Uganda in 1974 and has been frequent in Central Africa Region for the past 40 years. For over 40 years, Ebola was treated as an African disease, called a fever and known by other names where occurrences have been frequent. EVD has become a global public health threat following the most recent outbreak in West Africa. By December 31, 2014, Ebola has infected more than 23,500 people in West Africa and killed over 9,500, nearly all in the three worst-affected countries of Guinea, Liberia and Sierra Leone. It is transmitted through blood, vomit, diarrhea and other bodily fluids but cultural attributes associate its etiology to man-made and supernatural causes, hence stemming public health approaches to contain EVD difficult. Distrust and conflict between two healing systems are rife necessitating an African Model of EVD care and prevention. The African model remains indispensable to understand EVD and developing appropriate EVD containing approaches.

## Introduction

The current Ebola Virus Disease (EVD) outbreak began in Guinea in December 2013 and now involves intense transmission in Guinea, Liberia, and Sierra Leone [1]. On Friday 25th July 2014 the Director-General (DG) of the World Health Organization declared the Ebola outbreak to be a Public Health Emergency of International Concern (PHEIC) under the International Health Regulations (2005), and grading the outbreak to Grade 3 in line with the WHO Emergency Response Framework (ERF) [2]. There is heightened concern about the risk of Ebola spreading to other countries. Health officials are exploring ways to prevent similar disease outbreaks around the world but will have to grapple with underlying global perceptions and beliefs about Ebola. These underlying beliefs and perceptions may have been responsible for previous slow global response to the disease. We comment on the controversies around the history, naming, contextual and global action on EVD.

## History of EVD: globalization, conflicting history and conflicting names

The dates related to the exact year of the first outbreak of the Ebola Virus Diseases (EVD) have not been firmly established. Symptoms similar to the EVD were identified in 1967 at Marburg leading to the name Marburg Virus of the filoviradae virus family [3]. The tracing and quarantining of people who had been in contact with the victims was used to contain the spread of the virus [4]. Ebola is considered the second filovirus [3, 5]. Peters and LeDuc ascertain that the EVD started as a minor infection in Uganda in 1974 and has been frequent in Central Africa Region for the past 40 years. Others talk of 1972 and a few [5] about 1976. For over 40 years, Ebola was treated as an African disease, called a fever and known by other names in Uganda, The Republic of Congo, The Democratic Republic of Congo, Gabon and Ivory Coast where occurrences have been most frequent. Ebola Virus Disease (EVD) has become a global public health threat following the most recent outbreak in West Africa. Forty years after, Ebola has spread to Europe, America and Asia at smaller scale. Ebola has now been named a crisis requiring global actions [3, 5]. The name for Ebola has evolved from Ebola haemorrhagic fever to Ebola Virus Disease. It is a severe disease caused by a virus of the filovirus family, which occurs in humans and other primates. According to Peters and LeDuc the disease emerged in 1976 in almost simultaneous outbreaks in the Democratic Republic of the Congo (DRC) and Sudan. They explain that Ebola disappeared from Africa after 1979 and was only recognized again in 1994 [5]. Subsequent outbreaks have been occurring with increasing frequency. The largest outbreak to date is currently occurring in West Africa affecting primarily Guinea, Liberia and Sierra Leone [1] A small and unrelated outbreak was confirmed in the Democratic Republic of Congo in August 2014. Three smaller outbreaks occurred in 2012; 1 in the Democratic Republic of Congo, and 2 separate outbreaks in Uganda.

Peters and LeDuc have identified 5 species of Ebola virus, 4 of which have caused disease in humans [5]. These four include: Zaïre ebolavirus (EBOV), Sudan ebolavirus (SUDV), Tai Forest (TAFV) (formerly known as Ebola Ivory Coast) and Bundibugyo ebolavirus (BDBV). The fifth species, Reston ebolavirus (RESTV), has caused severe illness in non-human primates but not in humans. RESTV has more frequently occurred outside Africa. RESTV was first detected in October 1989 in Reston, Virginia (USA) in a colony of monkeys imported from the Philippines, and has subsequently caused outbreaks in non-human primates in Pennsylvania (Philadelphia), Texas (Alice) and Italy (Sienna). Several researchers and workers became infected with the virus during these outbreaks, but did not become ill. The current outbreak of EVD started in Guinea. Following the naming that is linked to the places or countries of origin, this species may be called Guinea Ebola, making up the sixth species. Scientists have made attempts to link the species to any previous species but have concluded that the Guinea Ebola species is unrelated to other previous ones. Table 1 below presents confirmed cases and outbreaks of Ebola virus disease from when it was first identified to 2014. The data from 2014 has been culled from WHO (2014) and Center for Disease Control (CDC 2014) [2, 6]. The current outbreak that started in 2014 continues. In Africa, outbreaks of EVD have historically primarily occurred in remote villages close to tropical rainforests in Central and West Africa. Confirmed cases of Ebola have been reported in the Democratic Republic of the Congo (DRC, formerly Zaire), Sudan, Gabon, Uganda, Republic of Congo, Cote d'Ivoire, and in Guinea, Liberia and Sierra Leone during 2014 [5]. During 2014, Ebola outbreaks have occurred for the first time in Guinea, Liberia and Sierra Leone, and in these countries there has been intense transmission in urban areas. Related to this extensive outbreak Ebola has been imported into Nigeria, Mali, Senegal, Spain, UK and the USA.

**Table 1:** Confirmed cases and outbreaks of Ebola virus disease to 2014 (data from WHO 2014)

Year	Country	Ebola virus species	No of cases	No of Deaths	Case fatality rate
1976	Zaire (Democratic Republic of Congo)	Zaire	318	280	88%
1976	Sudan	Sudan	284	151	53%
1976	England	Sudan	1	0	0%
1977	Zaire (DRC)	Zaire	1	1	100%
1979	Sudan	Sudan	34	22	65%
1989	USA	Reston	0(a)	0	0%
1990	USA	Reston	0(a)	0	0%
1992	Italy	Reston	0(a)	0	0%
1994	Gabon	Zaire	52	31	60%
1994	Côte d'Ivoire	Tai Forest	1	0	0%
1995	Democratic Republic of Congo	Zaire	315	254	79%
1996	Gabon	Zaire	31	21	57%
1996	Gabon	Zaire	60	45	75%
1996	South Africa	Zaire	1	1	100%
2000-1	Uganda	Sudan	425	224	53%
2001-2	Gabon and Republic of Congo	Zaire	124	97	78%
2002-3	Republic of Congo	Zaire	143	128	89%
2003	Republic of Congo	Zaire	35	29	83%
2004	Sudan	Sudan	17	7	41%
2005	Republic of Congo	Zaire	12	10	75%
2007	Democratic Republic of Congo	Zaire	264	187	71%
2007	Uganda	Bundibugyo	149	37	25%
2008	Philippines	Reston	0(b)	0	0%
2008-9	D R Congo	Zaire	32	14	47%
2011	Uganda	Sudan	1	1	100%
2012a	Uganda	Sudan	24	17	70%
2012b	Uganda	Sudan	7	4	40%
2012	D R Congo	Bundibugyo	57	29	55%
2014	Guinea, Liberia and Sierra	Guinea	23500	9500	40.5%
Total			25888	11090	67%

## West Africa in an Ebola outbreak context

EVD has affected populations and health workers equally, making everyone within the health system vulnerable and helpless. There has been untold social and economic impact of Ebola disease in Africa. Socially, there has been a strong bearing in the social integration of people. Stigma and discrimination of families, whole villages, tribal communities and countries have emerged as a norm [7, 8]. These happen because of the paucity of information, the belief systems and healing practices and the fear of unknown risk amongst the people. There have been a lot of changes and reactions among people and countries since the outbreak of Ebola virus. This occurs at several levels. Inside affected countries, families who have lost one of its members as a result of Ebola are perceived by other community members as potential source of infection and thus stigmatized and discriminated[1, 5] Significantly affected communities cannot move freely in their country either out of fear of discrimination or by rules put in place to govern affected people or communities. Among African states, we note that there is closure of boarders with affected countries, the imputation in popular discourse status Ebola patient to nationals of those countries, the distance and strict control of people from the affected states if ever the opportunity and obligation to stay in a country was necessary. Between Africa and the rest of the world, this disease, before it appeared in Europe and the USA was seen by a Western category as a disease of Sub-Sahara Africans. All these events express the idea that people have of the fatality of the disease and create a sense of shame and rejection of people, [7] a kind of dehumanization of man. These perceptions lead each other to focus attention on the distance and isolation as the only means to fight against the spread of the disease, yet many other useful preventive measures deserve to be known and incorporated. When there is an outbreak of an epidemic people initiate actions to stem its spread. As western societies introduced untested therapies to treat victims of Ebola, [5, 8] people in affected countries and those feeling vulnerable to the spread of Ebola equally initiated untested therapies grounded on their cultural ecology. In Benin, for example, people belief that the consumption of onion reduces the spread of Ebola. In Liberia, the adoption of salt by many communities as a therapeutic element prevailed for a while. Cameroonians and Nigerians also reacted by adopting the consumption of bitter cola and salt for the same reasons. A new untested dimension in Africa may be seeking help through prayer and from traditional healers. All of these popular culturally grounded strategies developed locally may not be able to reverse the upward trend of the disease on the continent. The technology and logistics used for tackling the Ebola Virus Disease looks far of reach to the African population, including human resources for health. The model and approaches are strange to even health professionals on the ground making them more vulnerable [1]. The health system is overstretched, ill-equipped and fragile to stand the challenge from Ebola. Health professionals fighting the Ebola epidemic respect a dress code, yet local populations co-opted or volunteering to assist in identifying and conveying the victims of Ebola to treatment centers go about the Ebola business almost without any special physical protection. The current models may be alienated, looking strange even to those who are purported to defend and uphold the public health system. This has given room to suspicion, scare and refusal of populations to adhere to the prescriptions of biomedical approaches of Ebola care [7] letting in the practice of traditional medicine [9] in a nearly domineering manner as with most disease outbreaks. Affected communities have been seeking ways linked to their cultures to contain the disease, yet the disease rages on. Curiously, the existing approaches may be paying little or no attention to the emerging cultural practices introduced by affected communities to contain the disease.

## Global concern, global action

Ever since Ebola was declared as a Global Epidemic, lots of global concerns have been raised and actions initiated. Within this framework, a WHO Ebola Response team is dedicated exclusively to coordinate international response, and mobilize international technical assistance from WHO, the Global Outbreak Alert and Response Network (GOARN) and other partners and stakeholders to provide direct support for national outbreak response activities and in the field [2]. The UN Security Council and the UN General Assembly, agreed on the establishment of the United Nations Mission for Ebola Emergency Response (UNMEER) by the UNSG - the first of its kind - headquartered in Accra and providing a UN-wide initiative that draws together all the assets of all relevant UN agencies to tackle this crisis together. In this regard, WHO is the lead agency for a number of strategic objectives? Travel alerts, travel restrictions and bans have been initiated to reduce the global impact that EVD may have [8]. Vaccines are being developed for trial and health protection and promotion measures have been introduced [8]. According to Media reports, the testing of the first vaccine has started in West Africa [5, 7] Global Consortiums have been set up to research and provide robust evidence that can facilitate the treatment of cases and the prevention of subsequent outbreaks.

## Conclusion

The uncertainty with which EVD has been reported and documented shows how neglected the disease has been. Less attention has been paid to EVD making it difficult for accurate forecasting to be done and the burden estimated. Soon, the EVD will diminish as the trend informs us now. When the Ebola crisis eventually begins to diminish, and the journalists and camera crews withdraw, public interest fades and political pressure on leaders subsides, the people of Sierra Leone, Liberia and Guinea will be left to rebuild their lives, their communities and their countries. Understanding the problems that led to the escalation of the Ebola crisis is essential in order for these countries to emerge safely from it and to prevent another crisis in the future. Systematic documentation of the EVD story and the attempts made to contain it may facilitate the initiation of future research and innovations. The research and innovations should respect the ethical guidelines and research regulations that would protect potential participants. There is some hope in containing EVD, as with other diseases, if an African model grounded on African culture is developed and tested. Such a model needs to consider the cultural ecology on which medicine, medical and public health practices survive in affected countries. Such models will reduce the wave of ongoing fear, stigma and discrimination that is stemming the current global efforts to eradicate the Ebola Virus Disease. An African model could possibly become an alarm and alert system developed with the people in need of protections.
